# Repurposing Itraconazole Loaded PLGA Nanoparticles for Improved Antitumor Efficacy in Non-Small Cell Lung Cancers

**DOI:** 10.3390/pharmaceutics11120685

**Published:** 2019-12-16

**Authors:** Nabil A. Alhakamy, Shadab Md

**Affiliations:** Department of Pharmaceutics, Faculty of Pharmacy, King Abdulaziz University, Jeddah 21589, Saudi Arabia

**Keywords:** chitosan, itraconazole, nanoparticles, lung cancer, apoptosis

## Abstract

Itraconazole (ITR) is a broad-spectrum antifungal drug, which has been shown to possess some promising anticancer, anti-proliferative, and anti-angiogenic properties in some cancers, such as cancers of the lung, breast, and skin. However, ITR has some drawbacks, such as poor water solubility, which hinder its use as a therapeutic agent. Therefore, in the present study, we developed and characterized chitosan-coated PLGA nanoparticles of itraconazole and studied their anticancer activities in H1299 lung cancer cells. The prepared ITR nanoparticles showed a small particle size, narrow poly dispersity index (PDI), positive zeta potential, and a controlled drug release profile. The cytotoxicity of ITR nanoparticles (NPs) on H1299 cancer cells after 24 h of exposure was greater than that of the ITR solution. Apoptosis of cancer cells exposed to ITR nanoparticles was also enhanced in comparison with the ITR solution. At the molecular level, ITR NPs were more effective than ITR solution in inducing pro-apoptotic Bax and p53 while reducing anti-apoptotic Bcl2 protein expression. ITR NPs were more effective than ITR solution in arresting cells both at the G0/G1 as well as G2/M phases of the cell cycle. Hence, repurposing itraconazole by encapsulation into PLGA NPs with chitosan coating is a potentially promising approach to treat lung cancers.

## 1. Introduction

Non-small cell lung cancer is one of the deadliest forms of cancer [1]; there is a critical need for an effective treatment of this disease. Itraconazole, a broad-spectrum, poorly water-soluble, and antifungal drug is ordinarily used for treating superficial infections caused by dermatophytes. The drug is active against a broad range of fungal infections, and is especially active against histoplasmosis, blastomycosis, *Aspergillus* app., and sporotrichosis [2]. Itraconazole’s anticancer effects include inhibition of angiogenesis, as well as other mechanisms, including inhibition of hedgehog signaling [3,4]. It has been suggested that a combination of itraconazole with an inhibitor of hedgehog signaling (GANT61) may be a novel approach for the prevention of breast cancer [4]. The drug was shown to possess potent antiangiogenic actions and to enhance the effectiveness of cytotoxic chemotherapy in multiple primary lung cancer xenografts [5]. The combination of itraconazole with standard chemotherapy in a small phase II study in patients with lung cancer showed an increase in overall and progression-free survival, and the authors speculated that the antiangiogenic properties of itraconazole might explain the positive effects [5].

The enhanced efficacy of itraconazole against human glioblastoma cancer cell lines by its incorporation into solid lipid nanoparticles with a coating layer of cationic lipid was reported previously [2]. Several platforms of nanoparticulates have been examined and formulated for the delivery of numerous drugs. Research has focused on nanoparticulate carriers that have particular qualities, relating to biodegradability, biocompatibility, enhanced bioavailability, and lack of toxicity [6]. The majority of new drugs are naturally hydrophobic or lipophilic and poor aqueous solubility militates against the use of lipophilic drugs because of the resultant low bioavailability; this can be overcome by their encapsulation in a nanoparticulate system. In order to deliver drugs in a controlled or sustained release manner, synthetic or natural biodegradable polymers are most commonly used. The major factors for the effective release of drug molecules from polymeric system are the molecular weight of the polymers, compatibility of the drug with the polymer, the mechanism of polymer degradation, and the solubility and permeability of the respective drug [7]. Among the several available biodegradable polymers, poly(lactic-*co*-glycolic acid) (PLGA) is widely used, as it has excellent degradation properties, is biocompatible, and can control or sustain the release of drugs [8]. Positively charged cationic polymers are used to increase membrane permeability and to promote intracellular drug absorption; they have the additional advantage that they can be customized according to the projected function requirement [8]. Chitosan, a naturally occurring, positively charged, biodegradable polymer, is used to improve the adhesion, interaction, and retention of nanoparticles at negatively charged mucosa or membranes, and to promote the diffusion of drug-loaded nanoparticles [9,10,11]. Repurposing ITR for inhibiting tumorigenesis in lung cancer in a nanoformulation is a novel approach. For this purpose, chitosan-coated ITR-loaded PLGA NPs were developed and characterized for their optimum particle size, particle size distribution, zeta potential, entrapment efficiency, and in vitro release. Furthermore, the antitumor activity of ITR and ITR nanoparticles was determined in H1299 lung cancer cells by examining cell viability, the presence of apoptosis, and cell cycle arrest; western blotting was used to examine the proteins involved in apoptosis.

## 2. Materials and Methods

### 2.1. Materials

ITR was purchased from Beta-Pharma (Shanghai, China). Poly(d,l lactic-*co*-glycolic acid; PLGA) (50/50, M.W. 45–70 kDa), medium molecular weight chitosan (CS), and tri polyphosphate (TPP) were purchased from Sigma-Aldrich, St. Louis, MO, USA. Β-actin (NB600-50155), Bax (NB100-609655), and Bcl2 (NB100-56098) primary antibodies were purchased from Novus Biologicals (Centennial, CO, USA). The apoptosis detection kit (556547) was purchased from BD Bioscience (NJ, USA). All other chemicals used were of analytical grade.

### 2.2. Development of Chitosan-Coated PLGA Nanoparticles

Single emulsion-sonication, using slight variations of a previously reported process [12], was employed for the preparation and optimization of itraconazole-loaded, chitosan-coated PLGA nanoparticles. The aqueous and organic phases were prepared separately. The aqueous phase consisted of 10 mL of CS solution (0.1% *w*/*v*) in 1% *v*/*v* glacial acetic acid containing 1% PVA and 1% Poloxamer-1880. The organic phase consisted of ITR (20 mg) together with 100 mg of PLGA (50:50) dissolved in dichloromethane (DCM; 1 mL). Dropwise addition of the organic phase into the aqueous phase was facilitated by means of a probe sonicator (SONICS Vibracell™, Sonics and Materials, Inc., (Newtown, CT, USA) for 3 min at 40% power. Emulsification was performed for 3 min with the whole assembly maintained on an ice bath. After the emulsification process, the mixture was placed on a magnetic stirrer at 500 rpm for 8 h at room temperature until the DCM was completely evaporated. Centrifugation of the formulation was undertaken for 40 min at 30,000 rpm at 4 °C. The pellet obtained was washed three times while the supernatant was collected and further used for analysis of the drug content. Milli Q water was used for the re-dispersion of the pellet, and the cryoprotectant (Trehalose 5%, *w*/*v*) was added before freeze-drying for 24 h to obtain freeze-dried, drug-loaded nanoparticles. The PLGA-NPs without CS coating were obtained by a similar process but without using CS and acetic acid in the aqueous phase.

### 2.3. Particle Size, Polydispersity, and Zeta Potential Measurement

Dynamic light scattering (DLS) and Laser Doppler Velocimetry (LDV) mode (Zetasizer Nanoseries-ZSP (Malvern Instruments, Worcestershire, UK) was used for the determination of the particle size, polydispersity index (PDI), and zeta potential of the developed nanoparticles. All experiments were performed in triplicate to give an average value and standard deviation for the particle size, PDI, and zeta potential.

### 2.4. Transmission Electron Microscopy and Scanning Electron Microscopy

The particle size and shape of CS-coated PLGA NPs was observed by transmission electron microscopy (TEM) (JEOL JEM1010, Tokyo, Japan). The diluted formulations were sonicated for 10 min and a drop of sonicated formulation was placed on a copper grid and allowed to completely dry the sample. After complete drying, the sample was viewed under TEM at an operating voltage of 80 kV. The surface morphology was observed by scanning electron microscopy (SEM) (Zeiss EVO LS10; Cambridge, UK) using the gold sputter technique.

### 2.5. Differential Scanning Calorimetry (DSC)

Physical state analysis of ITR, CS, PLGA, physical mixture (drug + polymers), ITR-PLGA NPs, and CS-ITR-PLGA NPs was conducted using a DSC-8000 (Perkin Elmer Instruments, Shelton, CT, USA). The results were analyzed using PYRIS V-11 software (Perkin Elmer Instruments, Shelton, CT, USA). The thermal behavior of the samples was performed at a scanning rate of 10 °C/min with the temperature range 30–300 °C.

### 2.6. Entrapment and Drug Loading Efficiency

Entrapment (%EE) and drug loading efficiency (%DL) were estimated as described by Kalam et al. [13]. In total, a 5-mL suspension of PLGA-NPs and CS-PLGA-NPs was ultracentrifuged at 4 °C, 30,000 rpm for 40 min. The obtained supernatants were analyzed for free ITR by the UPLC method as described by Kalam et al. [13]. The %EE and %DL were calculated through the following equations (Equations (1) and (2)):

(1) $$\%\mathrm{EE}=\left(\frac{\mathrm{Initial}\;\mathrm{weight}\;\mathrm{of}\;\mathrm{drug}\;\left(\mathrm{mg}\right)-\mathrm{Unencapsulated}\;\mathrm{weight}\;\mathrm{of}\;\mathrm{drug}\;\left(\mathrm{mg}\right)}{\mathrm{Initial}\;\mathrm{weight}\;\mathrm{of}\;\mathrm{drug}\;\left(\mathrm{mg}\right)}\right)\times 100$$

(2) $$\%\mathrm{DL}=\left(\frac{\mathrm{Initial}\;\mathrm{weight}\;\mathrm{of}\;\mathrm{drug}\;\left(\mathrm{mg}\right)-\mathrm{Uunencapsulated}\;\mathrm{weight}\;\mathrm{of}\;\mathrm{drug}\;\left(\mathrm{mg}\right)}{\mathrm{Total}\;\mathrm{weight}\;\mathrm{of}\;\mathrm{nanoparticles}\;\left(\mathrm{mg}\right)}\right)\times 100$$

### 2.7. In Vitro Release Study

The release study was carried in PBS (pH 7.4) containing sodium lauryl sulphate (0.5%, *w*/*v* solution) to improve the solubility of ITR, up to 72 h as per the reported methods [12,14]. The NPs and ITR solution in DMSO (all equivalent to 1.6 mg in 1 mL) were transferred into the dialysis membrane tubing (MWCO = 12 kDa), which were immersed in beakers containing 40 mL of release media, following which the beakers were placed in a shaking water bath at 37 ± 1 °C and 50 rpm. After predetermined time points (3, 4, 6, 8, 24, 48, and 72 h), samples were collected from the beakers, centrifuged at 13,500 rpm for 15 min, and analyzed by UPLC [13]. To ensure the sink condition, the same volume of fresh media was replaced after each sampling. All the formulations were subjected to in vitro release in a set of three (*n* = 3).

## 3. Cell Culture

The present work was carried out in human embryonic kidney cell line, HEK 293, and a NCI-H1299 non-small cell lung cancer (NSCLC) cell line. These cells were obtained from National Centre for Cell Science NCCS Pune, India. These cells were cultured in Dulbecco’s modification of eagle’s medium (DMEM) supplemented with 10% fetal bovine serum (FBS), 100 U mL^−1^ penicillin, and 100 mg mL^−1^ streptomycin at 37 °C in a 5% CO_2_ incubator with 95% humidity. The cells were subcultured when they achieved 80% to 90% confluence and used for experiments.

### 3.1. In Vitro Cell Viability Assay

The MTT assay was employed to evaluate the viability of ITR, ITR NPs, and blank CS-coated NPs-NPs in a lung cancer cells (H1299 cells). The procedure was performed by taking 96-well plates in which the cells were seeded independently at a density of 10,000 cells in each well having 100 μL of DMEM medium at 37 °C for 24 h. After culturing for 24 h, subsequently, the removal of media was undertaken and substituted with new medium having ITR solution, ITR NPs, and blank CS-coated PLGA NPs formulations at different concentrations (5–100 μg/mL). The plates were incubated at 37 °C in 5% CO_2_ for 24 h followed by 4 h of incubation after adding MTT solution (5 mg/mL) per well. The supernatant MTT solution was removed and 200 μL of DMSO was added to each well. The microplate reader was used to measure the absorbance at 570 nm. Cells treated with DMSO (0.1% *v*/*v*) diluted with culture medium were used as a negative control and considered to be 100% viable. The same procedure was carried out for human embryonic kidney cell line, HEK 293, cells to see the cytotoxic effect of ITR solution, ITR NPs, and blank CS-coated PLGA NPs formulations on a normal cell line. The formulations were tested in the same dose range as that tested for the non-small cell lung cancer cell line H1299.

### 3.2. Apoptosis Assay by Flow Cytometry

Dual staining with fluorescein isothiocyanate (FITC)-annexin V and propidium iodide (PI) kit (BD Biosciences PharMingen) was used to evaluate apoptosis by flow cytometry (BD FACS Aria^Tm^ III, NJ, USA). H1299 cancer cells were seeded in a 6-well plate at a density of 300,000 cells per well and incubated for 24 h. Cells were then treated with ITR and ITR NPs for 24 h. Successively, by the addition of trypsin, the cells were harvested and centrifuged for 5 min at 1500 rpm. The cells were then dispersed in 500 μL of binding buffer and further incubation was performed for 15 min with AnnexinV FITC (2 µL) and PI (5 µL). Quantification of apoptotic cells was performed by flow cytometry using a FACS DIVA 8.0.2 [15].

### 3.3. Effect of ITR and ITR NPs on Molecular Markers

Western blot analysis was performed to study the effect of ITR and ITR NPs on the expression of apoptotic markers (p53, Bax, and Bcl-2) in H1299 lung cancer cells. H1299 cells were seeded in 6-well plates and incubated with ITR and ITR NPs overnight as described previously by Zhao et al. [16]. The cells were washed twice with PBS and incubated in RIPA lysis buffer for 30 min on ice. Liquid-containing proteins were obtained by collecting the supernatant after centrifugation (12,000 rpm at 4 °C). The extracted proteins were separated using SDS-PAGE and transferred to PVDF membranes. The membrane was left in TBST with 5% BSA at room temperature for 2 h to block any remaining binding sites on the membrane. The membrane was incubated with primary antibodies to β-actin, p53, BAX, and Bcl-2. After 10 h, the membrane was then incubated with horseradish peroxidase-conjugated secondary antibodies for 1 h. The signal was detected using a chemiluminescence reagent kit and visualized protein bands quantification was performed by densitometric analysis using image J software (NIH, MD, USA).

### 3.4. Cell Cycle Status by Flow Cytometry

Cell cycle status for ITR and ITR NPs was assessed in the H1299 cell line as described by Zhang et al. [17]. H1299 cells treated with ITR and ITR NPs for 24 h were collected, pelleted, and washed twice with phosphate-buffered saline (PBS) and incubated with nuclei staining buffer (RNase A (7 kU/mL), and PI solution (50 μg/mL)) for 2 h. The cell cycle distribution was measured by using the flow cytometry using a FACS DIVA 8.0.2.

### 3.5. Statistical Analysis

The results are represented as average ± SD, and data were analyzed using Student’s *t*-test and one-way ANOVA followed by Tukey’s post hoc test (*n* = 3) to assess statistical significance (*p* < 0.05) using SPSS software.

## 4. Result and Discussions

### 4.1. Preparation and Characterization of Itraconazole Nanoparticles

Blank PLGA NPs (PLGA NPs), ITR-loaded PLGA NPs (ITR-PLGA NPs), and ITR-loaded PLGA NPs with chitosan coating (CS-ITR-PLGA NPs) were prepared by the single-emulsion ultrasonication method for encapsulating hydrophobic drugs. The main physicochemical characteristics of the prepared PLGA NPs, ITR-PLGA NPs, and CS-ITR-PLGA-NPs are shown in Table 1.

**Figure 1:**
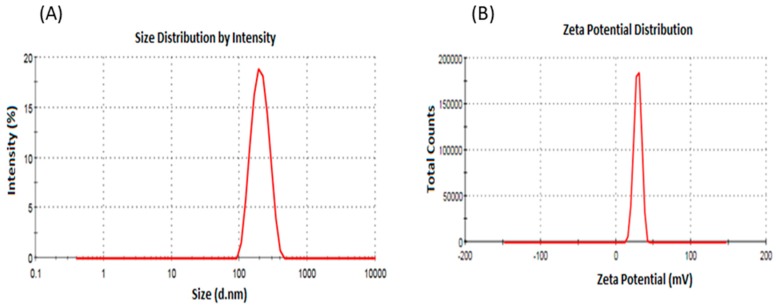
(**A**) DLS analysis: Hydrodynamic size and particle size distribution of CS-ITR-PLGA NP. (**B**) Zetasizer: Zeta potential of CS-ITR-PLGA NP.

**Figure 2:**
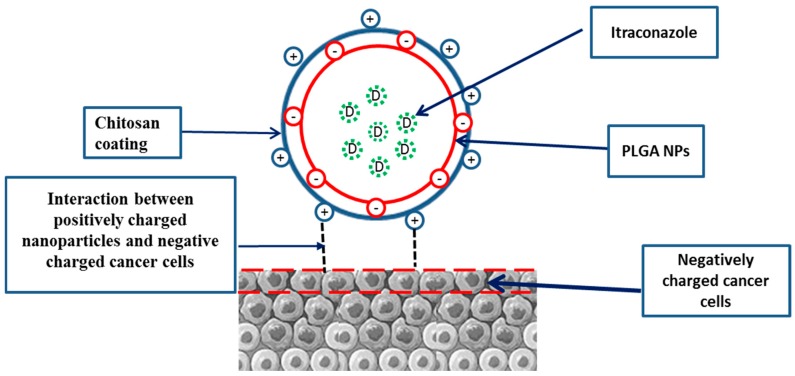
Schematic presentation of chitosan-coated PLGA NPs and their interaction with cancer cells. The positive surface of nanoparticles promotes adhesion and retention by cancer cells, which have negatively charged membranes.

The average particle size of PLGA NPs and ITR-PLGA NPs was found to be less than 200 nm with a good PDI. Loading of ITR into the NPs did not significantly alter the average particle size, PDI, or zeta potential (*p* > 0.05). The zeta potential value for both formulations was found to be low, and negative for PLGA NPs due to the presence of terminal carboxyl groups on the PLGA polymer producing a negative surface potential. The addition of CS led to a significant increase in particle size and ZP (*p* < 0.05). The increase in particle size of CS-ITR-PLGA NPs compared with PLGA NPs and ITR-PLGA NPs is due to increased viscosity of the chitosan-added aqueous phase; this lowers the shear stress during ultrasonication of the emulsion, and results in a larger particle size [18]. This agrees with the previous observations of Lima et al. [19], who developed ferulic acid-loaded PLGA nanoparticles with a chitosan coating. A small particle size is important in determining a better biodistribution, increased systemic stability, and enhanced permeability and retention [16]. The satisfactory PDI of CS-ITR-PLGA-NPs indicates a narrow, uniform, and homogenous distribution and successful development of the formulation (Figure 1A). The zeta potential of CS-ITR-PLGA NPs was found to be +21.13 ± 3.42 mV as compared to ITR-PLGA NPs (0.90 ± 0.55) (*p* < 0.05); this increased the stability of the NPs (Figure 1B). The increased positive zeta potential value is due to the presence of amino groups in the CS chain; this suggests that the ITR-PLGA NPs were sufficiently coated by CS. The positive surface charges of NPs also promote adhesion to and retention by cancer cells, which have negatively charged membranes (Figure 2) [20]. The CS coating reduces the chances of interaction of nanoparticles with phagocytes and their absorption by these cells, because positively charged nanoparticles are less readily absorbed [21].

**Figure 3:**
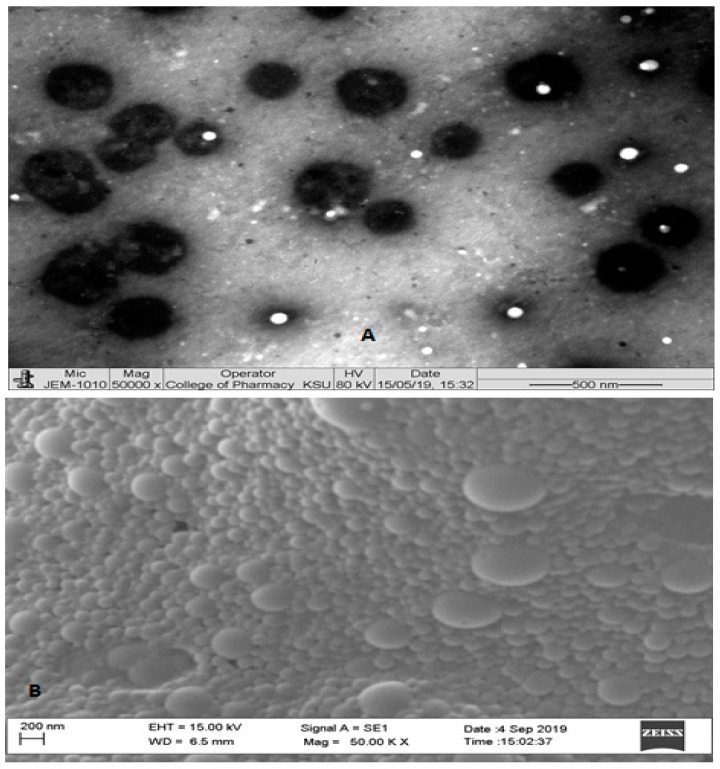
(**A**) TEM analysis: Actual size analysis of chitosan-coated ITR-loaded PLGA NPs. (**B**) SEM analysis: Surface morphology of chitosan-coated ITR-loaded PLGA NPs.

The entrapment efficiency of ITR-PLGA NPs and CS-ITR-PLGA NPs was found to be 79.68% ± 7.30% and 80.18% ± 8.12%, respectively, with respective drug loadings of 15.93% ± 1.46% and 16.03% ± 1.62%; thus, addition of the CS coating did not significantly alter the drug loading and entrapment efficiency (Table 1; *p* > 0.05). This was not in agreement with previous findings [19,21], which showed that CS-coated PLGA nanoparticles loaded with paclitaxel and ferulic acid increased the hydrophilicity of the system, resulting in decreased entrapment of hydrophobic drugs [19,21]. As shown using TEM, the particles were perfectly spherical, with the CS coating uniformly distributed on the surface (Figure 3A). The SEM images of CS-ITR-PLGA NPs showed a spherical, or slightly oval, morphology and agglomerated particles, resulting in an increase in the size (Figure 3B).

### 4.2. Differential Scanning Calorimetry (DSC)

**Figure 4:**
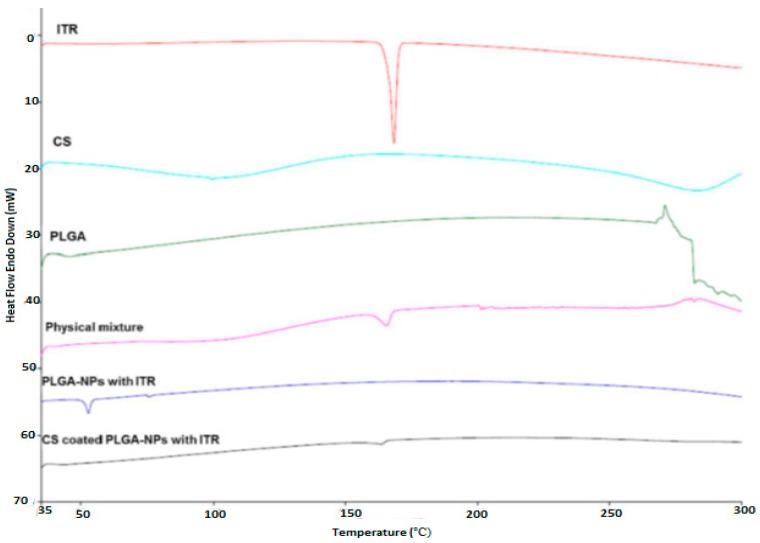
DSC of ITR, CS, PLGA, physical mixture (drug + polymer), ITR-PLGA NPs, and CS-ITR-PLGA NPs.

The comparative DSC patterns of ITR, CS, PLGA, physical mixture, ITR-PLGA NPs, and CS-ITR-PLGA NPs are shown in Figure 4. The DSC thermogram showed a sharp peak of ITR at 165.4 °C while CS showed a peak around 300 °C and PLGA had a glass transition temperature (Tg) of 45.30 °C. There was no change in the ITR peak in the physical mixture, indicating compatibility between ITR, PLGA, and CS. There was no melting peak in the ITR-PLGA NPs and CS-ITR-PLGA NPs formulation, which is an indication of complete entrapment of ITR in nanoparticles and ITR present in the non-crystalline state (Figure 2). This agreed with other published findings [12].

### 4.3. In Vitro Drug Release Study

**Figure 5:**
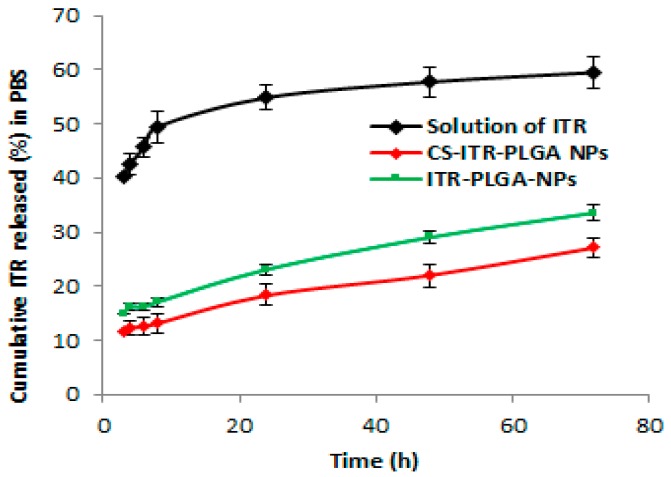
Cumulative in vitro release profiles of ITR, ITR-PLGA NPs, and CS-ITR-PLGA NPs formulations in phosphate buffer saline release medium at pH 7.4 over a period of 72 h. Data plots and error bars represent mean ± SD (*n* = 3).

A comparative drug release study of ITR solution, ITR-PLGA NPs, and CS-ITR-PLGA NPs was performed in pH 7.4 buffer at 72 h (Figure 5). ITR solution, ITR-PLGA NPs, and CS-ITR-PLGA NPs showed a drug release of 59.49% ± 4.24%, 33.56% ± 3.0%, and 27.12% ± 2.48%, respectively. The release study showed a significant controlled release pattern for ITR-PLGA NPs and CS-ITR-PLGA NPs as compared to ITR solution (*p* < 0.05), with an initial rapid release of ITR from PLGA NPs (16.18% ± 0.91%) and CS-ITR-PLGA NPs (12.32% ± 1.81%) at 4 h as compared to 72 h. The initial rapid release of ITR from PLGA NPs was reduced in chitosan-coated NPs. This may be due to the CS coating present on the NPs surface, which acts as an extra barrier for ITR release [22]. This release study agrees with the previous published literature for bevacizumab [23]. However, there was no significant difference between these formulations in the release of the drug at 4 h (*p* > 0.05). The initial rapid release and subsequent controlled release would maintain the effective concentration of ITR in PBS for a longer time. The drug release from polymeric nanocarrier systems involves several mechanisms. These include polymer degradation, erosion of the polymer, desorption from the particle surfaces, and diffusion, or a combination of all of these mechanisms [24,25]. Controlled release of ITR from formulations could be beneficial for the treatment of lung cancer.

### 4.4. Cytotoxic Effect/s of ITR and CS-ITR-PLGA NPs (ITR NPs)

**Figure 6:**
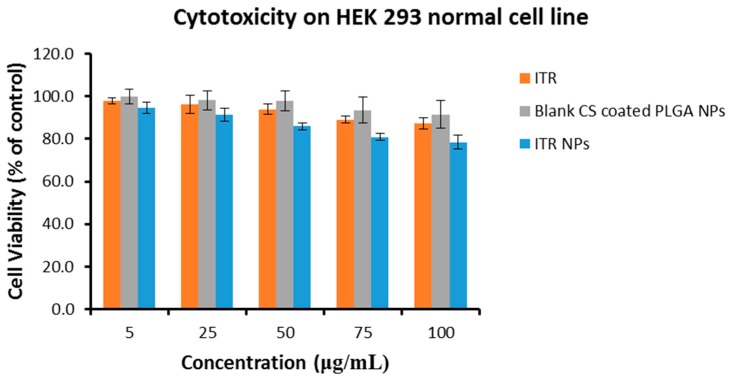
Cell viability of ITR solution, ITR NPs, and blank CS-coated PLGA NPs formulations on HEK 293 normal cells at 24 h. The cell viability was determined by MTT assay. Control consisted of cells treated with 0.1% DMSO (vehicle control, 100% cell viable). Data are expressed as mean ± SD (*n* = 3).

**Figure 7:**
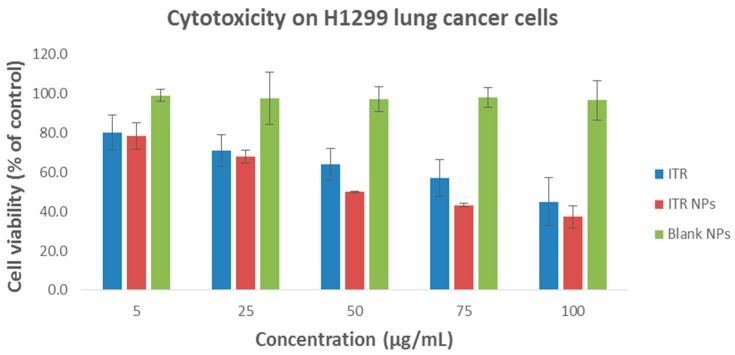
Cell viability of H1299 cancer cells after 24 h exposure of (A) ITR solution, ITR NPs, and blank CS-coated PLGA NPs formulations. The cell viability was determined by MTT assay. Control consisted of cells treated with 0.1% DMSO (vehicle control, 100% cell viable). Data are expressed as mean ± SD (*n* = 3).

Assays to study the effect/s of a natural or synthetic anticancer drug must include determination of both its safety to the host cells and its cytotoxic potential against cancerous cells [26]. The most widely used cytotoxicity test is the [3-(4,5-dimethylthiazolyl-2)-2,5-diphenyltetrazolium bromide] (MTT) assay. We evaluated the biosafety and/or cytotoxic effect of ITR solution, ITR NPs, and blank CS-coated PLGA NPs on human embryonic kidney cell line, HEK 293, which is the most widely used and readily available normal human cell line. The prepared formulations were tested in the same dose range as that tested for the non-small cell lung cancer cell line, H1299, and the cytotoxicity was minimal and non-significant (*p* ≤ 0.05) (Figure 6). Accordingly, the human non-small cell lung cancer cell line H1299 was used to study the effects of ITR and its nano-formulation (ITR NPs) on cell death and proliferation. At 24 h post-treatment, both ITR and ITR NPs produced a concentration-dependent response, as evidenced by the cell viability and/or toxicity against H1299 cells (Figure 7). At lower concentrations, the difference between ITR and ITR NPs in their effects on cell death was not significant while at higher concentrations (50–100 µg/mL), the difference was clear (Figure 7), with the cell death/cytotoxic effects of ITR NPs being more pronounced and significant (*p* ≤ 0.05) compared to ITR alone (Figure 7); this suggests a better cytotoxic potential of ITR when used as a nano-formulation. Further, the lower IC_50_ value of ITR NPs (63.17 µg/mL) compared to ITR (96.68 µg/mL) is also suggestive of a greater cytotoxic effect of the nano-formulation compared to the drug itself. The blank CS-coated PLGA NPs showed no toxicity at any concentration or time point (Figure 7). H1299 cancerous cells having higher anabolic demands respire predominantly by glycolytic means with a higher glucose/nutrient intake, lactate production, and high proliferation rate compared to non-cancerous cells, which makes them more exposed towards ITR/ITR NPs. ITR and ITR NPs showed less toxicity to HEK 293 normal cells as compared to H1299 cancer cells. The possible mechanism of ITR/ITR-NP showing higher toxicity to H1299 cancer cells could be due to interference with the nutrient uptake and selective inhibition of the membrane transporters in these cells, which are normally absent in non-cancerous cells. The greater cytotoxic effect of ITR NPs as compared to free ITR against H1299 cancerous cells could be attributed to the small size and rapid uptake of nano-formulations, which facilitates different mechanisms of transport, such as endocytosis or passive transport, into the cell through the lipid bilayer compared to the healthy cells. However, this is yet another aspect/field and needs a comprehensive evaluation at the molecular level to find the exact targets of these anticancerous formulations. All further experiments were carried out by using IC_50_ concentrations only. Taken together, the results are indicative of a better cytotoxic potential of ITR NPs than ITR alone against non-small cell lung cancer cells H1299.

### 4.5. Effect of ITR and ITR NPs on Cellular Apoptosis

**Figure 8:**
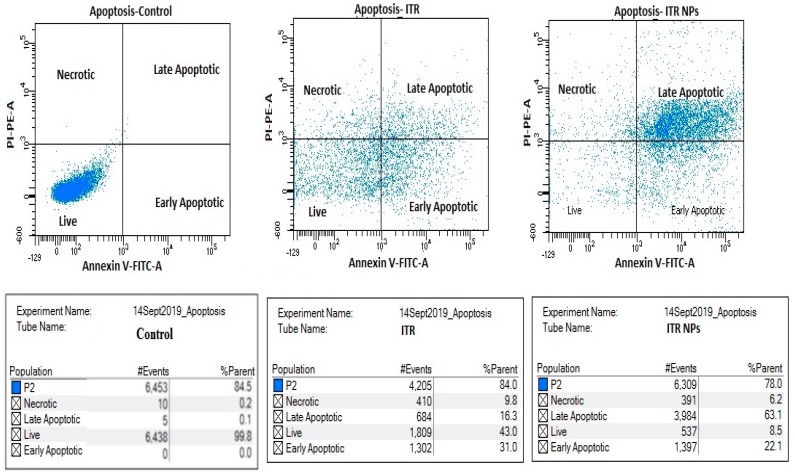
Flow cytometric dots plots analysis by propidium iodide (PI) in the *y*-axis and annexin V- Fluorescein isothiocyanate (FITC) in the *x*-axis staining of H1299 cells treated with the control group, ITR solution, and ITR NPs.

**Figure 9:**
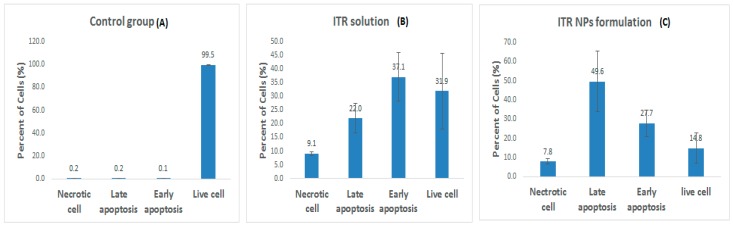
Quantitative data for induction of apoptotic cell death by the (**A**) control group (untreated H1299 cells), (**B**) H1299 cells treated by ITR solution and (**C**) ITR NPs. Bar graph analysis showing higher total apoptotic cell death (early + late apoptosis) induced by ITR NPs treatment compared to ITR solution. Data are expressed as mean ± SD (*n* = 3). The data were analyzed using one-way ANOVA followed by Tukey’s post hoc test. The percentage of apoptotic cells in each group was compared with the control group, *p* < 0.05 indicates a significant difference.

To study the mechanism of the cytotoxic effects of ITR and its nano-formulation, we evaluated cell apoptosis post-drug treatment by flow cytometry, using an annexin V/FITC kit. ITR (96.68 µg/mL/24 h) significantly induced cellular apoptosis compared to the control group (Figure 8); when H1299 cells were treated with ITR NPs (63.17 µg/mL/24 h), the extent of apoptosis was more significantly (*p* < 0.05) increased compared to both the control as well as ITR-treated groups. However, ITR and ITR NPs both increased the number of necrotic cells number compared to the control group, although the percentage of necrotic cells of the latter was slightly lower than the former (Figure 9). Further, the percentage of early and late apoptotic cells in the ITR (52.1%)-treated group was significantly lower (*p* < 0.05) than the ITR-NPs (76.9%)-treated group, suggesting a strong apoptosis-inducing potential of the nano-formulation (Figure 9). The lower percentage of early apoptotic cells in the ITR NPs-treated cells (26.6%) than in those treated with ITR alone (37.1%) indicates that the former has strongly and quickly rendered cancerous cells to apoptosis compared to the latter as evident from the percentage of late apoptotic cells from the two groups (Figure 9).

**Figure 10:**
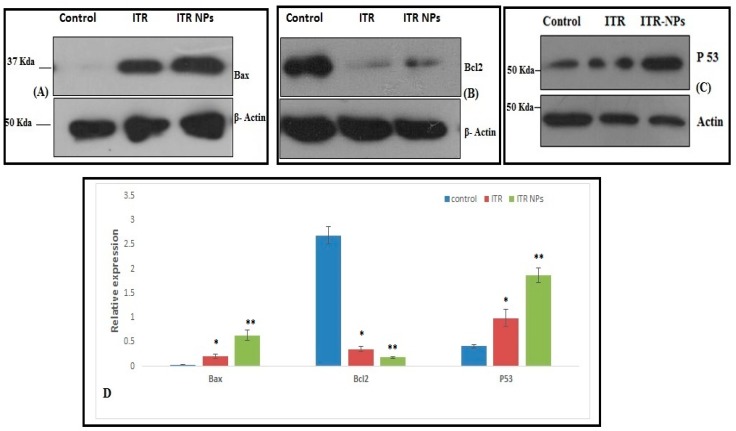
Represent the effect of ITR and ITR NPs on the expression pattern of (**A**) BAX, (**B**) Bcl2, and (**C**) p53 proteins in H1299 cells through western blot analysis. β-actin was taken as the loading control. (**D**) Densitometric analysis of the above bands representing expression of Bax and Bcl2 was performed using image j software. *p* < 0.05 was considered to indicate a statistically significant difference (* ITR vs. ITR NPs; ** control vs. ITR NPs).

We evaluated the effect of ITR and its nano-formulation at the molecular level by studying the expression of the pro-apoptoptic protein p53, Bax, and the anti-apoptotic protein Bcl2 using western blotting (Figure 10). Treatment with ITR and ITR NPs significantly upregulated the expression of the pro-apoptotic protein p53 and Bax compared to the control group (*p* ≤ 0.01); however, the effect was much more pronounced in the ITR NPs-treated group as compared to ITR alone (*p* ≤ 0.05) (Figure 10). A significant increase in the p53 and Bax expression is consistent with the higher cell cytotoxic effect of ITR NPs in H1299 cancer cells. Similarly, the expression of the anti-apoptotic protein Bcl2 was significantly reduced in both ITR and ITR NPs-treated groups compared to the control group (*p* ≤ 0.01); again, the reduction was more pronounced with ITR NPs than with ITR alone (*p* ≤ 0.05) (Figure 10). Taken together, these observations suggest a greater apoptotic effect of ITR NPs than ITR alone on non-small cell lung cancer cells H1299. Apoptosis is a programmed cell death mechanism destined to remove the cells with extensive DNA damage, which otherwise could lead to various deleterious effects, including cancer [27]. Apoptotic evasion is an established hallmark of cancer; hence, triggering apoptosis in cancerous cells could be an important aspect in cancer therapy [28]. Previous reports have suggested that ITR induced cell death via apoptosis induction and/or autophagy in breast cancer cells [29]. Apoptosis could be intrinsic or extrinsic, both of which ultimately converge through the executioner caspases as the final pathway [30]. Annexin V (AV) is a Ca^2+^-dependent phospholipid-binding protein, which has a higher affinity for phosphatidyl serine (PS) found towards the inner cytosolic side of the cell membrane. Four types of cells could be found after annexin V FITC/PI staining: Live, necrotic, early, and late apoptotic cells [31]. It can be speculated that both ITR and ITR NPs activated the intrinsic apoptotic pathway. This is supported by the fact that we observed a clear upregulated expression of the pro-apoptotic protein, p53, Bax and a decrease in the anti-apoptotic counterpart, Bcl2 [32] in ITR and ITR NPs-treated groups compared to the control. Such a change in p53, and Bax/Bcl2 expression is reported to cause cytochrome C release from mitochondria, which in turn activates caspases-9 and hence caspases-3 to initiate apoptosis [33,34,35]. Likely, this could be one attribute of the mechanism/s responsible for increased cell death and increased apoptosis by ITR or its nano formulations ITR NPs; however, additional studies are needed to establish this mechanism of action more comprehensively.

### 4.6. Effect of ITR and ITR NPs on Cell Cycle Progression

**Figure 11:**
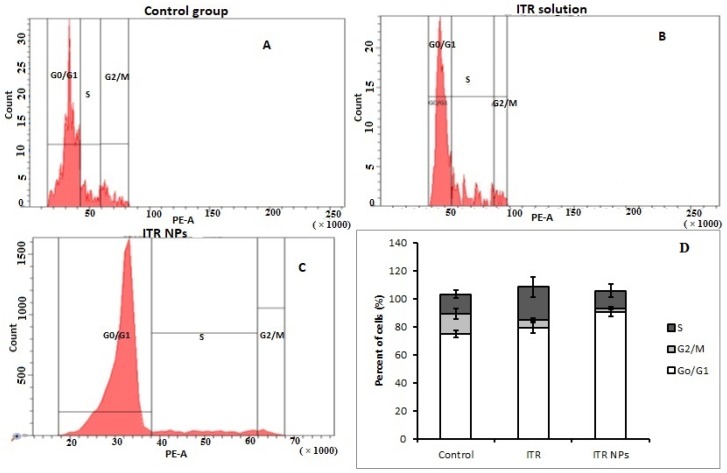
Flow cytometric analysis of the effect of (**A**) control group, (**B**) ITR and (**C**) ITR NPs in H1299 lung cancer cells at 24 h of cell cycle arrest. (**D**) Bar graph analysis showing percent of cells in different phases of cell cycle. The data were analyzed using one-way ANOVA followed by Tukey’s post hoc test. Percentage of viable cells in each group was compared with VC, *p* < 0.05 indicates a significant difference.

To investigate whether apoptotic induction is related to cell cycle arrest, the ITR and ITR NPs-treated cells were analyzed using PI (a DNA binding dye). It was observed that treatment with both ITR and ITR NPs significantly reduced the proportion of cells in the G2/M phase, thus indicating inhibition of cell division compared to the control group (Figure 11A–D). Further, both ITR and its nano-formulation arrested the cells in the G0/G1 phase, although the percent increase in cell arrest was not significant in ITR (79.56% ± 3.78%)-treated cells; however, the ITR NPs treatment significantly increased the proportion of cells in the G0/G1 phase (90.53% ± 2.87%) compared to both the control (74.90% ± 2.74%) as well as ITR (79.56% ± 3.78%)-treated groups (Figure 11D). The increase in the percentage of cells in the S phase in the ITR-treated group was probably due to the fact that such cells were actively synthesizing DNA; however, they failed to enter the G2/M phase, perhaps because of reduced ATP levels and a higher energy demand, causing the cells to undergo necrosis. This suggested an alternative mechanism of inducing cell death by ITR. Overall, the results suggest that both ITR and ITR NPs significantly inhibited cell cycle progression, with the effects being more pronounced in the latter group. Uncontrolled proliferation characterized by a deregulated cell cycle is one of the most prominent cancer hallmarks [31], and inducing cell cycle arrest is a desirable trait for most anticancer drugs. In a stimulated phase, cancer cells tend to remain in a state of active growth and division characterized by a continuous transition between G1 to S to G2 to M phases of the cell cycle. However, the cell cycle is regulated by checkpoints, G1/S (at cells tending to enter the synthesis phase) and G2/M (cells tending to enter the active mitotic phase). The G1/S checkpoint is the limiting step in the cell cycle and if the cancer cells are stopped at this stage, the sustained proliferative tendencies could be abrogated effectively. In the present study, we observed an increase of G0/G1 cells, with this increase being more significant in ITR NPs-treated cells compared to cells treated with ITR alone; this suggests a greater potential of the nanoformulation to decrease sustained proliferation. However, neither ITR nor ITR NPs could significantly reduce/arrest the S phase; this is indicative of an alternative cell death pathway, as evident from necrotic populations in both ITR as well as ITR NPs-treated groups. The significant decline in the proportion of cells in the G2/M phase of ITR NPs-treated groups as compared to ITR is indicative of G2/M phase arrest; these results are in agreement with some previous reports [36,37].

## 5. Conclusions

We successfully developed and evaluated chitosan-coated ITR-loaded PLGA nanoparticles as a potential treatment for lung cancer. The prepared nanoparticles showed an acceptable particle size, narrow particle size distribution, spherical shape, high entrapment efficiency, and controlled drug release. Compared with ITR alone, ITR NPs showed higher cytotoxic, and apoptotic cell death in the H1299 cell line, with cells being arrested both at the G0/G1 and G2/M phases of the cell cycle. ITR NPs were more effective than ITR solution in inducing pro-apoptotic Bax, p53 protein while reducing the expression of the anti-apoptotic protein, Bcl2. Based on these findings, we suggest that repurposing itraconazole by its encapsulation into chitosan-coated PLGA NPs could be an interesting approach to treat lung cancers.

## Figures and Tables

**Table 1:** Average particle size, polydispersity index, zeta potential, entrapment efficiency, and drug loading of blank PLGA NPs, ITR-PLGA NPs, and CS-ITR-PLGA-NPs.

Formulations	Average PS (nm) ± SD	Average PDI ± SD	Average ZP (mV) ± SD	Average EE (%) ± SD	Average DL (%) ± SD
PLGA NPs	189.3 ± 3.4	0.086 ± 0.06	−4.04 ± 1.21	NA	NA
ITR-PLGA NPs	184.53 ± 4.43	0.18 ± 0.06	0.90 ± 0.55	79.68 ± 7.30	15.93 ± 1.46
CS-ITR-PLGA-NPs	275.9 ± 6.09	0.24 ± 0.074	21.13 ± 3.42	80.18 ± 8.12	16.03 ± 1.62

NA: not applicable. SD: standard deviation; Particle size (PS); Polydispersity index (PDI), Zeta potential (ZP); Entrapment efficiency (EE) and drug loading (DL).
